# Learning to ignore salient color distractors during serial search: evidence for experience-dependent attention allocation strategies

**DOI:** 10.3389/fpsyg.2013.00326

**Published:** 2013-06-19

**Authors:** Adam T. Biggs, Bradley S. Gibson

**Affiliations:** Department of Psychology, University of Notre DameNotre Dame, IN, USA

**Keywords:** salience, visual selection, visual attention, dilution, perceptual load

## Abstract

Previous research has investigated whether visual salience (i.e., how much an item stands out) or perceptual load (i.e., display complexity) is the dominant factor in visual selective attention. The evidence has been mixed, with some findings supporting a dominant role for visual salience and some findings supporting a dominant role for perceptual load. However, the complex displays used to impose high perceptual load also introduce a third factor that has gone understudied until recently: the interplay between identity dilution and exposure duration. Adding display items to increase perceptual load dilutes a distractor's identity, which could decrease interference, but the task generally takes longer, which could increase distractor interference. To clarify how these factors interact, the present study used converging measures of distractor interference—both compatibility and singleton presence—to disambiguate effects due to salience, perceptual load, and identity dilution/exposure duration. Compatibility effects support perceptual load as the dominant factor, whereas singleton presence effects do not (Experiment 1). Consistent with salience-based mechanisms, significant distractor processing (both compatibility and presence effects) occurred under high perceptual load when singleton present trials preceded singleton absent trials (Experiment 2A). However, consistent with load-based mechanisms, non-significant compatibility effects occurred under high perceptual load when singleton absent trials preceded singleton present trials (Experiment 2B). Thus, the competition between salience-based and load-based mechanisms depended on the amount of prior experience with singleton present vs. absent displays, which in turn depended on the use of broad vs. narrow attentional allocation strategies. These experience-dependent effects provide further evidence that attention allocation strategies are contingent on factors such as task context and experience.

Visual selective attention enables observers to focus on goal-relevant information in the presence of irrelevant information. Currently, perceptual load theory (Lavie, 1995) is the leading view of visual selection, but this perspective has come under attack from a number of different sources. Two alternative accounts suggest that visual salience (e.g., Eltiti et al., 2005) or distractor dilution (e.g., Tsal and Benoni, 2010) describe visual selection better than perceptual load. However, distinguishing between these issues is often complicated as they tend to be manipulated through similar means, such as how adding heterogeneous letters to increase display size both reduces form-related salience and dilutes the critical distractor (Biggs and Gibson, in press). The present study will disambiguate these theoretical interpretations by providing multiple measures: singleton distractor presence effects will better correspond to salience, and singleton distractor compatibility effects will better correspond to dilution. Furthermore, we can explore how these bottom-up factors of salience and dilution interact with the top-down experience of the observer. In so doing, the present experiments will clarify the theoretical interpretation obtained from different patterns of distractor interference, and allow us to confidently advance our understanding of the factors that influence the efficiency of visual selective attention.

According to the *perceptual load account* of visual selection (Lavie and Tsal, 1994; Lavie, 1995, 2005, 2006; Lavie and Cox, 1997; Lavie et al., 2004), the ability to ignore distractors depends critically on what perceptual load the display imposes on an observer. This is because the extent to which critical distractors are processed depends on the extent to which perceptual capacity is consumed by the set of relevant items (i.e., potential target and non-target items). When the display is relatively small, relevant information does not consume all available resources and perceptual resources spill over to the distractor, typically resulting in significant distractor processing, or late selection. When the number of relevant items is relatively large, perceptual resources do not spill over to the distractor because they are fully consumed in processing the relevant items, typically resulting in non-significant distractor interference, or early selection. This idea remains the predominant view of visual selection as it appears to settle the long standing debate of early vs. late selection (Broadbent, 1958; Deutsch and Deutsch, 1963) by suggesting that the locus of selection is not fixed.

However, recent evidence has suggested that perceptual load theory may not be as comprehensive in explaining visual selection as once believed. Such evidence has tended to come in one of two forms. The first is in disrupting the empirical hallmark of perceptual load, where significant distractor interference occurs under low load and is eliminated or reduced under high load. Some evidence has challenged load theory by showing non-significant interference under low load (e.g., Paquet and Craig, 1997) or significant interference under high load (e.g., Biggs and Gibson, 2010; Cosman and Vecera, 2010). The second means of challenging load theory is through an alternative explanation as to why the empirical pattern arises in the first place (e.g., Tsal and Benoni, 2010). In either case of disputing evidence, other factors were shown to influence visual selection above and beyond the account of perceptual load theory. Two of these factors, particularly as the visual display is concerned, are salience and dilution.

According to the *visual salience account* of visual selection (Theeuwes, 1991, 1992, 1994; Theeuwes and Burger, 1998; Eltiti et al., 2005) the ability to ignore distractors depends critically on the relative salience of the distractor in question. This is supposedly because focal attention can be captured in a reflexive fashion by salient objects and events in the world. In this view, significant distractor interference should be observed regardless of the number of relevant items because the salience of a uniquely-colored distractor remains high across any load manipulation. Previous studies have placed load and salience in direct competition to better understand the relative contributions of each factor to visual selection, but the results have been mixed (Gibson and Bryant, 2008; Biggs and Gibson, 2010). On the one hand, some evidence suggests that load can dominate salience when low and high load displays are presented randomly, as reflected by a significant decrease in distractor interference for high load vs. low load trials (Gibson and Bryant, 2008; Biggs and Gibson, 2010, Experiment 1). On the other hand, some evidence suggests that salience can dominate load when low and high load displays are presented in separate blocks, as reflected by equal amounts of distractor interference observed across display load (Biggs and Gibson, 2010, Experiment 2).

The latter results, suggesting that salience can dominate load when knowledge of load increases (i.e., when high- and low-load displays are presented in separate blocks of trials), appear inconsistent with findings that salience effects typically decrease as display knowledge increases (Lamy and Yashar, 2008; Müller et al., 2009; but see, Pinto et al., 2005). Although, the fact that visual salience has any effect at all during the performance of this serial search task might be considered surprising. This is because observers typically adopt a relatively narrow focus of attention during these tasks and the computation of visual salience is thought to require a relatively wide focus of attention (Belopolsky et al., 2007). Because this pattern of distractor interference appears to lead to a puzzling conclusion, it is possible that the distractor interference observed by Biggs and Gibson (2010, Experiment 2) does not reflect capture by salience, but rather the operation of some other mechanism.

Another such mechanism affecting these various results is the dilution of distractor interference (Benoni and Tsal, 2010; Tsal and Benoni, 2010; Wilson et al., 2011). According to this alternative dilution account, there are two opposing factors that may be confounded with manipulations of load. One factor involves the dilution of distractor identities by other display items (see also Benoni and Tsal, 2010; Wilson et al., 2011). For example, a common manipulation of perceptual load is display size (e.g., Lavie, 1995; Lavie and Cox, 1997), but the addition of multiple non-target identities decreases (or “dilutes”) the amount of interference the distractor identity is capable of generating. High perceptual load then produces less distractor interference due to the potency of the critical distractor, not a difference in visual selection. The second factor involves exposure duration. When exposure duration is not controlled, it usually increases as a function of perceptual load because task performance is more difficult under conditions of high load than under conditions of low load. Increases in exposure duration, in turn, typically cause increases in distractor interference because observers are exposed to the distractors for longer periods of time (Gibson et al., 2009).

The magnitude of distractor interference observed in a given condition may reflect a combination of the offsetting effects through both dilution and exposure duration. In particular, when load is low, there is relatively weak dilution of the distractor's identity (which should result in increased distractor interference), but exposure duration is relatively short (which should result in decreased distractor interference). In contrast, when load is high, there is relatively strong dilution of the distractor's identity (which should result in decreased distractor interference), but exposure duration is relatively long (which should result in increased distractor interference). Thus, Biggs and Gibson (2010, Experiment 2) may have observed equal amounts of distractor interference across their manipulation of load not because the salient distractor consistently captured focal attention, but rather because the shifting balance between dilution and exposure duration that occurred with a change in perceptual load resulted in a constant amount of distractor processing.

If this alternative account of Biggs and Gibson's (2010, Experiment 2) findings is plausible, then visual salience may actually have little effect on visual selection in this paradigm. However, a primary issue is that these results rely upon an empirical pattern of distractor interference obtained from distractor compatibility effects, which potentially have multiple explanations. For example, non-significant distractor compatibility effects under high perceptual load could mean a lack of perceptual resources for processing, a non-salient distractor incapable of attracting sufficient resources to induce processing, or substantial distractor dilution. Another source of evidence is required to help delineate some of these possibilities.

This converging evidence can be provided by using different methods to measure distractor interference—singleton distractor presence and singleton distractor compatibility. In the present study, singleton distractor presence will be manipulated by whether a singleton distractor ring is present or absent from the display, whereas singleton distractor compatibility will be manipulated by altering the identity of a singleton distractor to be incompatible, neutral, or compatible with the target letter. These different manipulations provide two advantages to the present study. The first is that they easily map onto the factors of salience and dilution. Salience can correspond to the presence (or absence) or a color singleton distractor, and interference can be measured by the difference between response times when the distractor is present vs. when it is absent. Dilution can correspond to the potential conflict between target and distractor identities, which can be measured by the difference in response times when an incompatible vs. neutral distractor is present. Moreover, using a color singleton distractor allows it to remain salient whenever present, which circumvents an issue with distractor dilution. For example, if display size is the only manipulation in a letter search, then the increase in non-target letters to increase display size both dilutes the distractor and reduces its salience. The Gibson and Bryant (2008) paradigm allows us to include both salience and dilution manipulations because it includes a color singleton distractor, which can be made present or absent, and a letter appears inside the color singleton ring, which can be made incompatible, neutral, or compatible with the target identity. Our critical distractor can thus remain equally salient under both low and high perceptual load despite the introduction of additional non-target letter identities. Another advantage is that these different measures of distractor interference provide different insights into cognitive processing. Singleton distractor presence can measure the extent to which a particular item impacts attention, whereas singleton distractor compatibility effects can measure the extent to which the same item is processed. Thus, if any particular factor (e.g., perceptual load, visual salience, distractor dilution) is capable of fully explaining visual selection, its effects should be revealed across multiple measures.

## Experiment 1

Studies using the additional singleton paradigm (e.g., Theeuwes, 1991, 1992) have routinely manipulated the presence vs. absence of a salient distractor to measure attentional capture. In these studies, observers searched for a form singleton while also attempting to ignore an irrelevant color singleton. The critical results showed that RTs were significantly slower when the color singleton was present in the display relative to when it was absent. These results were interpreted as follows: in the distractor absent condition, observers searched for the form singleton, which they were able to detect efficiently regardless of display load. However, although observers also detected the form singleton efficiently regardless of display load in the distractor present condition, they were overall slower because attention was first shifted to the more salient color singleton before it was redirected to the relevant form singleton, thus causing a constant increase in search time. Furthermore, manipulations of distractor compatibility have also provided evidence for the notion that focal attention is shifted to the location of the irrelevant color singleton because significant distractor compatibility effects are typically observed in the additional singleton paradigm as well (Theeuwes et al., 2000). In short, manipulations of both singleton distractor presence and singleton distractor compatibility can lead to complementary findings within the context of the additional singleton paradigm, with the former manipulation typically resulting in a search-time cost and the latter manipulation typically resulting in interference due to the distractor identity.

Inclusion of a singleton distractor presence manipulation in the serial search paradigm used by Gibson and Bryant (2008; also Biggs and Gibson, 2010), in addition to the distractor compatibility manipulation, has the potential to provide converging evidence for any conclusion seeking to explain differences in distractor interference under varying conditions of perceptual load. Accordingly, our experimental manipulations include both singleton distractor presence and distractor compatibility under conditions of both low and high perceptual load. Note that consistent with Biggs and Gibson's Experiment 2, observers had full knowledge of perceptual load in the present experiment by virtue of the fact that low and high load displays were presented in separate blocks.

If the pattern of distractor interference observed in the low and high load conditions of Biggs and Gibson's Experiment 2 resulted from attentional capture, then singleton distractor presence should have some effect on the dynamics of visual search. In particular, based on previous evidence (Theeuwes, 1991, 1992), there is reason to expect that singleton distractor presence should result in a search-time cost and the distractor compatibility manipulation should continue to result in distractor interference because attention is routinely shifted to the salient color distractor first. In contrast, we can assess exposure to the singleton distractor and dilution through a comparison between the singleton distractor presence and compatibility manipulations. The role of exposure to the distractor can be assessed by singleton distractor presence, which should result in significant search time costs, but it can be dissociated from dilution, which should result in non-significant distractor interference effects under high perceptual load as the additional non-target letters will dilute the distractor. If the presence of the singleton distractor has no effect and we only observe differences in distractor compatibility effects, then perceptual load theory offers the best explanation of the results.

### Method

#### Participants

Twenty-three undergraduate students from the University of Notre Dame participated in the experiment for partial completion of a course requirement. Data from two observers were removed due to an error rate over 20%, and one for not complying with the instructions (i.e., continually responding as though the singleton distractor identity were the target letter). All observers reported normal or corrected-to-normal visual acuity without color deficits.

#### Stimuli and apparatus

The search displays were similar to those used by Biggs and Gibson (2010). The items appearing in the target display were placed at equal intervals on an imaginary circle with radius of 3.2° visual angle at a viewing distance of 57 cm. Each item was a letter subtending 0.76° in height and 0.55° in width surrounded by a ring with diameter of 1.4°. The rings were either green (18.81 cd/m^2^) or red (18.81 cd/m^2^), but the letters were always gray (18.48 cd/m^2^). In the four-item displays, the items were placed at the four cardinal locations, and in the 12-item displays, two additional items were placed in between each of these cardinal locations. In the distractor absent condition, all the rings had the same color (red or green). In the distractor present condition, all but one of the rings had the same color; the remaining “singleton distractor ring” appeared in the opposite color. The commonly-colored rings always contained one of the two target letters (*E* or *R*) and a variable number of non-target letters (*H, P, N, K*, or *F*). When the singleton distractor ring was present, it contained a letter that was equally likely to be incompatible, neutral (*T*), or compatible with respect to the target's identity. When the singleton distractor was absent, the extra commonly-colored ring that replaced the singleton distractor ring always contained the letter *T* (as in the neutral condition). Responses were measured with a custom-made button box (Lafayette Instruments) and recorded to the nearest millisecond. Timing and presentation of stimuli were controlled by the DMDX experimental software program (Forster and Forster, 2003).

#### Procedure

**Figure 1:**
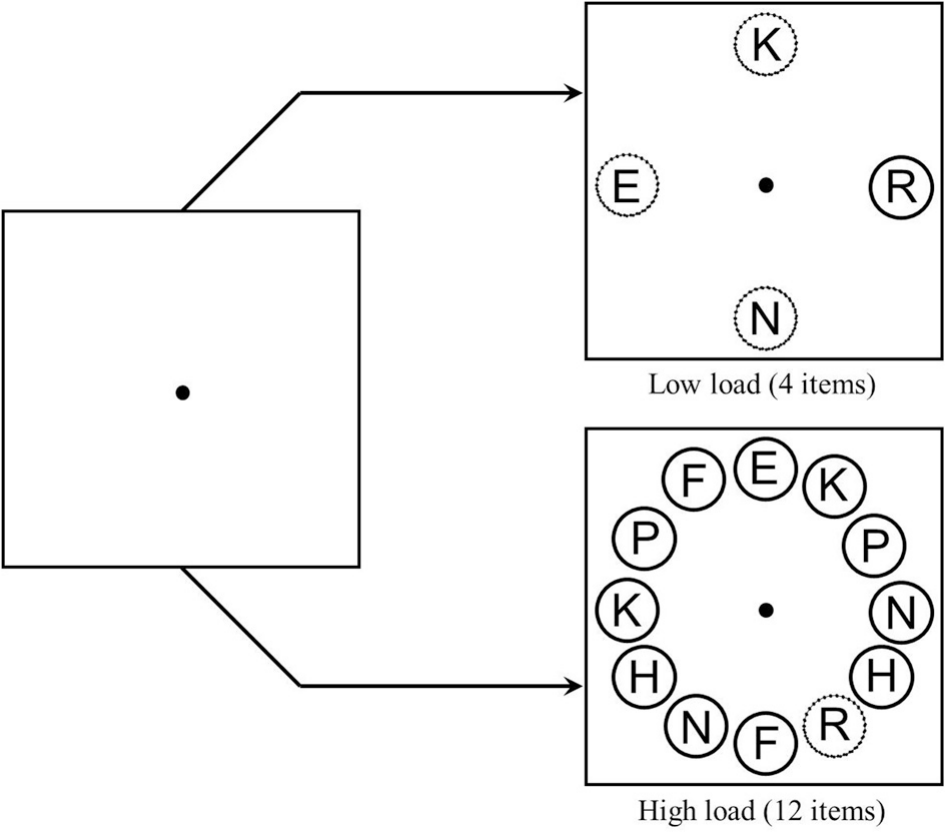
**Sample trial sequence**. Each trial began with a fixation display for 500 ms, followed by the experimental display which remained on screen until a response was made. Solid lines represent green circles, and dotted lines represent red circles as used in the actual experiment.

See Figure 1 for a sample trial sequence. A fixation dot appeared for 500 ms and was followed by the target display. Observers were instructed to keep their eyes on the fixation dot and search among the commonly-colored rings for the target while ignoring any uniquely-colored rings in the display. Observers were instructed to press the left key as quickly and as accurately as possible when they thought the target display contained the *R*, and they were instructed to press the right key as quickly and as accurately as possible when they thought the target display contained the *E*. The target display remained visible until response or until 4 s elapsed.

Observers had full knowledge of color conditions and perceptual load on any given trial in the present experiment. More specifically, each observer was exposed to only one of the two possible color assignments for the target/non-targets and distractor rings during the experimental session. For example, a given participant would see red target and non-target rings throughout the entire experiment with a green singleton distractor ring, whereas another participant would see the reverse assignment. The two color assignments were counterbalanced across observers. Upon answering, the computer proceeded automatically to the next experimental trial. Observers completed a practice block of 12 trials before each perceptual load condition. Half of the experimental trials were singleton distractor present trials, and half were singleton distractor absent trials; there were 864 total experimental trials. Order of load condition viewed first (low/high or high/low) was also counterbalanced across participants.

### Results

**Figure 2:**
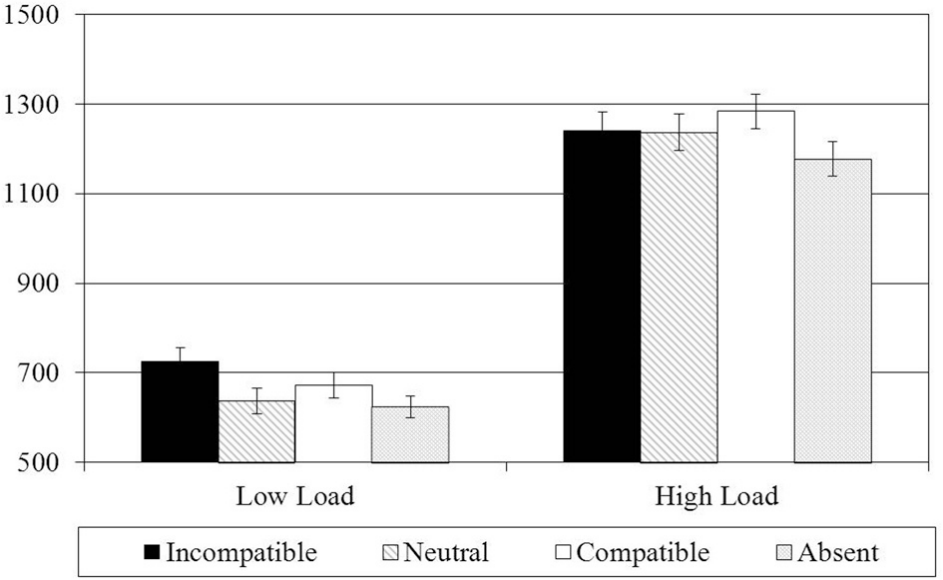
**Mean RTs (in ms) shown as a function perceptual load and distractor condition in Experiment 1**. Error bars reflect the standard error of the mean.

Note that incorrect key responses, response latencies greater than 4000 ms (the time limit of the experiment), or response latencies less than 200 ms (considered anticipatory and not intentional responses to specific targets) were treated as errors and excluded from the RT analyses in this and all subsequent experiments reported with this study (note that less than 1% of the data were excluded based on the two response latency criteria). Analyses focused on two measures of distractor processing: singleton distractor compatibility and singleton distractor presence. The analysis of singleton distractor compatibility provided a measure of distractor processing (incompatible distractor condition [I] - neutral distractor condition [N]); and, the analysis of singleton distractor presence provided a measure of the cost of systematically attending to the singleton distractor (neutral distractor condition [N] - singleton distractor absent condition [A]). Because the same experimental data is examined through both singleton distractor compatibility and singleton distractor presence, we will correct for the multiple comparisons with a Bonferonni correction, making our critical *p* value for significance 0.025 (α/2). Mean correct RTs are shown in Figure 2 as a function of perceptual load and distractor condition. Corresponding error rates are listed in Table 1.

**Table 1:** **Percent error rates listed as a function of perceptual load and distractor condition in Experiment 1**.

	**Distractor condition**			
	**Incompatible**	**Neutral**	**Compatible**	**Absent**
Low load	7.30 (1.80)	3.48 (0.85)	3.55 (0.64)	3.02 (0.82)
High load	4.72 (0.84)	4.26 (0.73)	7.51 (1.38)	4.06 (0.72)

(Standard errors appear in parentheses).

#### Singleton distractor compatibility

A 2 × 2 within-subjects ANOVA was conducted on mean correct RTs with perceptual load (low load vs. high load) and singleton distractor compatibility (incompatible vs. neutral) as the two within-subjects variables. There was a significant main effect of perceptual load, *F*_(1, 19)_ = 286.79, *p* < 0.001, η^2^_*p*_ = 0.94, with faster RTs in the low load condition (680 ms) than in the high load condition (1238 ms); and, there was also a significant main effect of singleton distractor compatibility, *F*_(1, 19)_ = 17.67, *p* < 0.001, η^2^_*p*_ = 0.48, with RTs in the incompatible distractor condition (982 ms) being slower than RTs in the neutral distractor condition (937 ms). Most importantly, however, these results were qualified by a significant perceptual load × singleton distractor compatibility interaction, *F*_(1, 19)_ = 12.17, *p* < 0.01, η^2^_*p*_ = 0.39, which indicated a larger distractor compatibility effect in the low load condition [I – N = 87 ms, *t*_(19)_ = 7.72, *p* < 0.001] than in the high load condition [I – N = 3 ms, *p* > 0.5].

An identical within-subjects ANOVA was performed on error rates. There was a significant main effect of distractor compatibility, *F*_(1, 19)_ = 7.77, *p* < 0.025, η^2^_*p*_ = 0.29, with fewer errors committed in the neutral distractor condition (3.87%) than in the incompatible distractor condition (6.01%). The interaction between these two variables approached significance *F*_(1, 19)_ = 3.66, *p* = 0.071, η^2^_*p*_ = 0.16, indicating a larger singleton distractor compatibility effect in the low load condition [I – N = 3.82%, *t*_(19)_ = 2.57, *p* < 0.025] than in the high load condition [I – N = 0.47%, *p* > 0.5]. Hence, these findings do not compromise the RT findings reported above.

#### Singleton distractor presence

A 2 × 2 within-subjects ANOVA was conducted on mean correct RTs with perceptual load (low load vs. high load) and singleton distractor presence (present vs. absent) as the two within-subjects variables. There was significant main effect of perceptual load, *F*_(1, 19)_ = 290.86, *p* < 0.001, η^2^_*p*_ = 0.94, with faster RTs in the low load condition (631 ms) than in the high load condition (1207 ms); and, there was also a marginally significant main effect of singleton distractor presence, *F*_(1, 19)_ = 17.72, *p* < 0.001, η^2^_*p*_ = 0.48, with RTs in the neutral distractor condition (937 ms) being slower than RTs in the singleton distractor absent condition (901 ms). Most importantly, however, these results were qualified by a significant perceptual load × singleton distractor presence interaction, *F*_(1, 19)_ = 7.59, *p* < 0.025, η^2^_*p*_ = 0.29, which indicated a smaller singleton distractor presence effect in the low load condition [N − A = 13 ms, *t*_(19)_ = 1.53, *p* = 0.14] than in the high load condition [N − A = 59 ms, *p* < 0.001]. An identical within-subjects ANOVA was performed on error rates. There were no significant main effects or a significant interaction in the error rates (all *F*s < 1).

### Discussion

Based on singleton distractor compatibility effects, we observe the typical perceptual load results where significant interference under low perceptual load was eliminated under high perceptual load. The singleton distractor remained salient in both low and high load conditions, yet its identity does not seem to have been processed. This evidence appears to support a perceptual load account of distractor processing. However, we observed the reverse for singleton distractor presence effects as the interference was non-significant under low load and significant under high load. Salience could explain why the singleton distractor presence effects were stronger under high perceptual load if we assume that, since all items are processed under low perceptual load anyway, salience is a much more important issue for the limited processing available under high perceptual load. The more complex high load displays were also on-screen longer due to the more complicated search, which indicates a difference in the amount of exposure to the critical distractor. The idea of exposure to the distractor is an intriguing one, particularly if we expand the notion to consider exposure as the amount of exposure to singleton distractors across the entire experiment instead of exposure during an individual trial. Some evidence does suggest that distractor rejection can depend upon prior experience (Leber and Egeth, 2006a,b; Vatterott and Vecera, 2012), which would indicate that exposure is more important to consider across the experiment rather than during a single trial or only under high perceptual load.

**Figure 3:**
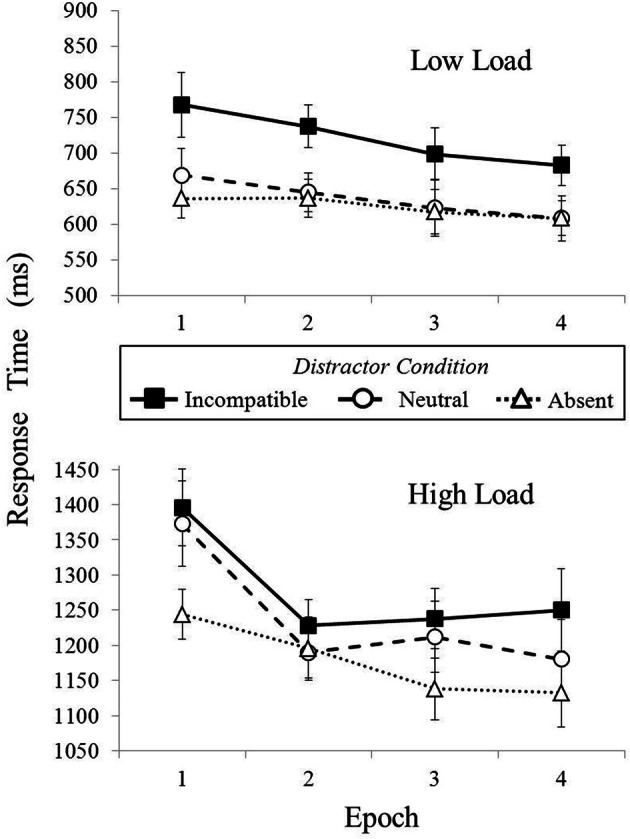
**Mean RTs (in ms) shown as a function perceptual load and distractor condition in Experiment 1**. Epoch represents when during the experiment the trial occurred as divided into quarters. Error bars reflect the standard error of the mean.

To address this issue, we divided up the low and high load trials into four blocks each based upon when the observed encountered a given trial during the experiment (leaving over 100 trials occurring in each block for each perceptual load condition). The results are shown in Figure 3. Under low load, the division seems to support the observations made from overall singleton distractor presence and compatibility effects. Singleton distractor presence seems to make very little difference as the singleton distractor neutral and absent trials were almost identical across the entire experiment. However, under high perceptual load, interference caused by the presence of a singleton distractor varies significantly. Singleton distractor presence was significant in the first block [N − A = 129 ms, *t*_(19)_ = 2.89, *p* < 0.01] and approached significance in the third block [N − A = 74 ms, *t*_(19)_ = 2.36, *p* < 0.05], but was non-significant in the second block [N − A = −5 ms, *p* > 0.50] and fourth block [N − A = 48 ms, *t*_(19)_ = 1.36, *p* = 0.19]. The change in the presence effect from the first to second block is largely due to the dramatic drop in response times when the singleton distractor was present, but thereafter the incompatible distractor condition begins to plateau. Continued decline in the singleton distractor absent condition, and some variance in the distractor neutral condition, is responsible for the differences in the latter portion of the experiment.

The observer appears to adopt a strategy sufficient to overcome distractor processing (e.g., block 2) only to see its effects short-lived. So, the exposure or experience of the observer appears to matter more under high load than low load, but it is difficult to make any specific argument for a learned strategy. Arguing for successful suppression of the distractor seems inappropriate given the variance in presence effects, yet with the singleton distractor appearing at random intervals, the only concrete conclusion is that exposure to the singleton distractor across the experiment impacts attention. A more controlled measure of singleton distractor presence is required to say more.

## Experiment 2

Experiment 1 appears to better support a dilution account of visual selection rather than the alternative explanations of perceptual load and salience. More importantly, exposure to the distractor seems to be a highly influential aspect, especially when we consider exposure as an experiment-wide issue rather than a within-trial issue. This highlights the potential impact of experience-dependent aspects of attention, which alter how an individual processes the world based upon their previous interactions with specific stimuli. For example, distractor interference can vary as a function of individual exposure to the distractor, and whether that particular observer has been able to develop an effective distractor suppression strategy (Müller et al., 2009; Zehetleitner et al., 2012). These examples involve specific distractors that the observer learns to filter out, which saves people from random city sounds and annoying siblings; all possible through a learned process of sustained attentional suppression (Dixon et al., 2009; Kelley and Yantis, 2009).

These experience-dependent issues raise two relevant questions for the present investigation. First, are these mechanisms only important for high perceptual load where strategy and salience appear to have the largest impact? If so, then effective means of suppressing distractors under low perceptual load require some other means of directing attention, such as a valid spatial cue (Johnson et al., 2002). If not, then experience-dependent mechanisms are important for both low and high perceptual load. The second question involves why there is a change in distractor processing. Increased distractor processing appears to occur when the task context biases the observer toward processing the distractor (Biggs and Gibson, 2010), but experience-dependent mechanisms all appear to revolve around using distractors to either better filter information (Leber and Egeth, 2006a,b; Dixon et al., 2009; Vatterott and Vecera, 2012) or to use distractors to more effectively guide attention, as with contextual cuing (Chun and Jiang, 1998, 1999). Salient distractors appear to require more effort to successfully filter out from processing, which suggests that experience-dependent mechanisms are providing the observer with a more effective filter.

However, an alternative is that salient distractors are successfully ignored when the observer becomes better at allocating attention to relevant stimuli; this idea essentially says that the best way to truly ignore something is to focus on something else. Consider the attentional white bear phenomenon (Tsal and Makovski, 2006), where being told to ignore a distractor results in the observer allocating attention to it. Any effort to suppress something, even one which develops effectively over time, still requires an effort. So, the more effective method of ignoring distractors may simply be to pay them no attention whatsoever and prioritize attention only to the relevant stimuli.

Using the same paradigm as Experiment 1, we will test this possibility by blocking distractor presence rather than randomly presenting the distractor. If observers are developing better mechanisms of filtering the distractor, then blocked distractor presence should enhance their opportunity to develop and employ any such filter. The result would be decreased distractor interference through singleton distractor presence or singleton compatibility effects. However, if this ability depends upon more efficient allocation of attention only to relevant target information, then it will require practice with singleton distractor absent rather than singleton distractor present circumstances. This finding would be supported by a difference that depends on whether or not a block of singleton distractor absent trials preceded the singleton distractor present trials. Only when a block of singleton distractor absent trials appear first would the observer have the time to appropriately learn to attend to the relevant target information, which would allow them to ignore a salient distractor simply by attending to something else rather than putting effort into active suppression.

## Experiment 2A

### Method

#### Participants

Seventeen undergraduate students from the University of Notre Dame participated in the experiment for partial completion of a course requirement. All observers reported normal or corrected-to-normal visual acuity without color deficits.

#### Stimuli and apparatus

Identical to Experiment 1.

#### Procedure

Each observer was exposed to four separate blocks of trials in this experiment. Half of the observers saw this order: high load, distractor present; high load, distractor absent; low load, distractor present; low load, distractor absent. The other half of the observers saw this order: low load, distractor present; low load, distractor absent; high load, distractor present; high load, distractor absent. Note that blocks of distractor present trials always preceded blocks of distractor absent trials within each load condition in Experiment 2A (see Experiment 2B for additional manipulations). There were 216 experimental trials presented in each of the four blocks (864 total experimental trials) and a representative set of practice trials preceded each block. A different random order of experimental trials was presented to each observer within each block.

### Results

**Figure 4:**
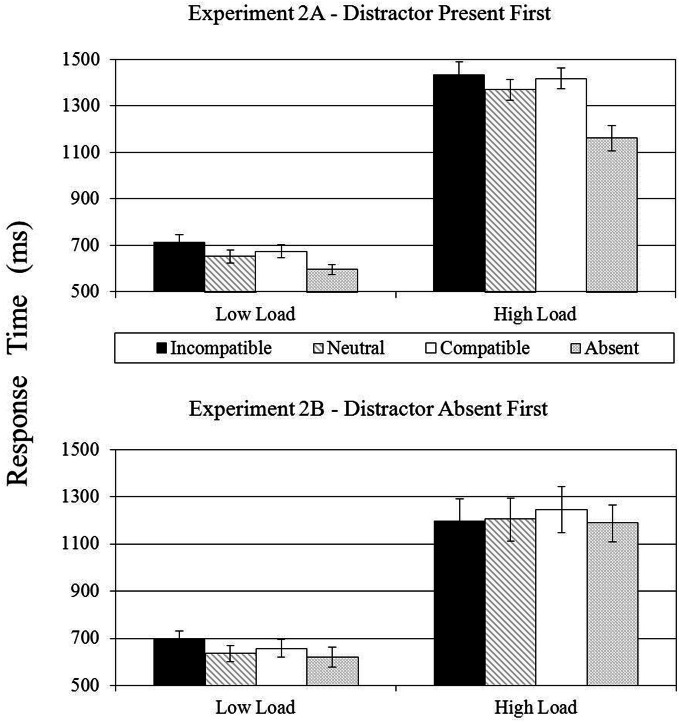
**Mean RTs (in ms) shown as a function perceptual load and distractor condition in Experiments 2A (top panel) and Experiment 2B (bottom panel)**. Error bars reflect the standard error of the mean.

Mean correct RTs are shown in the top panel of Figure 4 as a function of perceptual load and distractor condition. Corresponding error rates are listed in Table 2.

**Table 2:** **Percent error rates listed as a function of perceptual load and distractor condition in Experiments 2A and 2B**.

	**Distractor condition**			
	**Incompatible**	**Neutral**	**Compatible**	**Absent**
**Experiment 2A**			
Low load	5.22 (0.99)	2.18 (0.58)	3.48 (0.69)	3.06 (0.67)
High load	5.90 (1.04)	5.66 (1.08)	8.33 (1.96)	4.58 (0.67)
**Experiment 2B**			
Low load	3.69 (0.59)	2.95 (0.64)	2.87 (0.89)	3.37 (0.65)
High load	5.81 (1.18)	5.41 (1.08)	5.81 (1.12)	4.01 (0.80)

(Standard errors appear in parentheses).

#### Singleton distractor compatibility

A 2 × 2 within-subjects ANOVA was conducted on mean correct RTs with perceptual load (low load vs. high load) and singleton distractor compatibility (incompatible vs. neutral) as the two within-subjects variables. As expected, there was a significant main effect of perceptual load, *F*_(1, 16)_ = 201.31, *p* < 0.001, η^2^_*p*_ = 0.93, with faster RTs in the low load condition (683 ms) than in the high load condition (1401 ms). Also as expected, there was a significant main effect of singleton distractor compatibility, *F*_(1, 16)_ = 24.10, *p* < 0.001, η^2^_*p*_ = 0.60, with RTs in the incompatible distractor condition (1074 ms) being slower than RTs in the neutral distractor condition (1010 ms). Most importantly, consistent with the findings reported by Biggs and Gibson (2010, Experiment 2), the interaction between these two variables did not approach significance (*F* < 1), suggesting that the salient distractor captured focal attention under both low and high perceptual load. An identical within-subjects ANOVA was performed on error rates, but neither the main effects nor the interaction approached significance (all *F*s < 1). Thus, the RT results do not appear to be compromised by a speed-accuracy trade-off.

#### Singleton distractor presence

A 2 × 2 within-subjects ANOVA was conducted on mean correct RTs with perceptual load (low load vs. high load) and singleton distractor presence (present vs. absent) as the two within-subjects variables. As expected, there was a significant main effect of perceptual load, *F*_(1, 16)_ = 213.03, *p* < 0.001, η^2^_*p*_ = 0.93, with faster RTs in the low load condition (624 ms) than in the high load condition (1264 ms). More importantly, consistent with the attentional capture interpretation of the singleton distractor compatibility effect, there was also a significant main effect of singleton distractor presence, *F*_(1, 16)_ = 61.92, *p* < 0.001, η^2^_*p*_ = 0.80, indicating a 133 ms search-time cost in the singleton distractor present condition relative to the absent condition. Note, however, that these main effects were qualified by a significant perceptual load × singleton distractor presence interaction, *F*_(1, 16)_ = 38.64, *p* < 0.001, η^2^_*p*_ = 0.71. Although the pattern of singleton distractor compatibility effects reported above suggested that the color singleton captured attention equally well across the low- and high load conditions, this interaction indicated that the presence of the singleton slowed search more in the high load condition [N − A = 208 ms, *p* < 0.001] than in the low load condition [N − A = 58 ms, *t*_(16)_ = 3.84, *p* < 0.001], perhaps because the color singleton appeared more salient when it appeared among 11 commonly-colored items than when it appeared among three commonly-colored items. At any rate, this significant interaction is contrary to the predictions of the perceptual load account.

An identical within-subjects ANOVA was performed on error rates. Neither the main effects, nor the interaction approached significance (all *F*s < 1). Thus, the RT results do not appear to be compromised by a speed-accuracy trade-off.

### Discussion

Consistent with previous evidence (Biggs and Gibson, 2010, Experiment 2), the present study suggested that color salience can dominate perceptual load by providing converging evidence through both singleton distractor presence and singleton distractor compatibility. Moreover, the search-time cost associated with singleton distractor presence was larger in high load than low load, which might reflect the larger role of salience in the high load condition. Thus, the consistent effect of singleton distractor compatibility observed in the present study does not appear to arise as result of the interplay between distractor dilution and exposure duration.

## Experiment 2B

The consistent effects of singleton distractor compatibility and presence observed in Experiment 2A were interpreted to reflect the capture of focal attention by the salient distractor. Recall that Biggs and Gibson (2010) considered this pattern of results to be unusual given that the effect of distractor compatibility observed in the high load condition of their experiments increased as observers' knowledge of display load increased, whereas the effect of distractor compatibility observed in the low load condition remained constant regardless of observers' knowledge of display load. Our previous work interpreted the change in the magnitude of the distractor compatibility effect from one knowledge context to the other as reflecting the operation of top-down strategies, even though most previous evidence has suggested that attentional capture by salient singletons typically decreases as task knowledge increases (e.g., Lamy and Yashar, 2008; Müller et al., 2009).

However, another possibility is that rather than developing effective distractor suppression strategies, the task context is actually biasing the observer toward processing the distractor. An alternative means of ignoring the distractor is effectively allocating attention to relevant information rather than suppressing irrelevant information. Experiment 2B was therefore conducted to determine if the irrelevant singleton distractor would have less effect on performance if it was only encountered after experience with singleton distractor absent trials. If the order in which the irrelevant singleton was encountered is important, then the magnitude of the singleton distractor compatibility and presence effects should be significantly reduced in the high load condition. Such evidence would be important because it would corroborate the notion that these effects are not driven in a purely stimulus-driven fashion in this paradigm, and may shed light on the nature of the attention allocation strategies that observers use to modulate the effects of distractor salience.

### Method

#### Participants

Eighteen undergraduate students from the University of Notre Dame participated in the experiment for partial completion of a course requirement. Data from two observers were removed due to an error rate over 20%. All observers reported normal or corrected-to-normal visual acuity without color deficits.

#### Stimuli and apparatus

The search displays were identical to Experiment 2A.

#### Procedure

The procedure was identical to Experiment 2A, with the sole exception being that blocks of distractor absent trials always preceded blocks of distractor present trials within each load condition in Experiment 2. Half of the observers saw this order: high load, distractor absent; high load, distractor present; low load, distractor absent; low load, distractor present. The other half of the observers saw this order: low load, distractor absent; low load, distractor present; high load, distractor absent; high load, distractor present.

### Results

Mean correct RTs are shown in the bottom panel of Figure 4 as a function of perceptual load and distractor condition. Corresponding error rates are shown in Table 2.

#### Singleton distractor compatibility

A 2 × 2 within-subjects ANOVA was conducted on mean correct RTs with perceptual load (low load vs. high load) and singleton distractor compatibility (incompatible vs. neutral) as the two within-subjects variables. There was a significant main effect of perceptual load, *F*_(1, 15)_ = 58.34, *p* < 0.001, η^2^_*p*_ = 0.80, with faster RTs in the low load condition (666 ms) than in the high load condition (1200 ms); and, there was also a marginally significant main effect of singleton distractor compatibility, *F*_(1, 15)_ = 4.02, *p* = 0.063, η^2^_*p*_ = 0.21, with RTs in the incompatible distractor condition (947 ms) being slower than RTs in the neutral distractor condition (919 ms). Most importantly, however, these results were qualified by a significant perceptual load X singleton distractor compatibility interaction, *F*_(1, 15)_ = 6.65, *p* < 0.025, η^2^_*p*_ = 0.31, which indicated a larger compatibility effect in the low load condition [I – N = 62 ms, *t*_(15)_ = 8.29, *p* < 0.001] than in the high load condition [I − N = −7 ms, *p* > 0.5].

An identical within-subjects ANOVA was performed on error rates. There was a significant main effect of perceptual load *F*_(1, 15)_ = 13.30, *p* < 0.01, η^2^_*p*_ = 0.47, with fewer errors committed in the low load condition (3.70%) than in the high load condition (5.78%); and, there was also a significant main effect of singleton distractor compatibility, *F*_(1, 15)_ = 6.47, *p* < 0.025, η^2^_*p*_ = 0.30, with fewer errors committed in the neutral distractor condition (3.92%) than in the incompatible distractor condition (5.56%). The interaction between these two variables was also marginally significant *F*_(1, 15)_ = 3.55, *p* = 0.079, η^2^_*p*_ = 0.19, indicating a larger compatibility effect in the low load condition [I − N = 3.04%, *t*_(15)_ = 3.58, *p* < 0.01] than in the high load condition [I − N = 0.24%, *p* > 0.5]. Hence, these findings do not compromise the RT findings reported above.

Contrary to the findings reported in Experiment 2A, the present findings suggest that perceptual load dominated salience when observers did not encounter the salient singleton until the second (and fourth) block of trials. Further evidence for the conclusion that the salient distractor had a different effect on visual search in the high load condition across Experiments 2A and 2B was sought by conducting a 2 × 2 mixed ANOVA on mean correct RTs with singleton distractor compatibility (incompatible vs. neutral) as the sole within-subjects variable and experiment (Experiment 2A vs. 2B) as the sole between-subjects variable. This analysis revealed a marginally significant singleton distractor compatibility × experiment interaction, *F*_(1, 31)_ = 4.22, *p* = 0.05, η^2^_*p*_ = 0.12, indicating that the compatibility effect was significantly larger in the high load condition of Experiment 2A (66 ms) relative to the high load condition of Experiment 2B (−7 ms).

#### Singleton distractor presence

A 2 × 2 within-subjects ANOVA was conducted on mean correct RTs with perceptual load (low load vs. high load) and singleton distractor presence (present vs. absent) as the two within-subjects variables. There was significant main effect of perceptual load, *F*_(1, 15)_ = 99.92, *p* < 0.001, η^2^_*p*_ = 0.87, with faster RTs in the low load condition (627 ms) than in the high load condition (1196 ms). However, neither the main effect of singleton distractor presence, nor the interaction between perceptual load and singleton distractor presence approached significance (both *F*s < 1).

An identical within-subjects ANOVA was performed on error rates. There was a significant main effect of perceptual load *F*_(1, 15)_ = 18.83, *p* < 0.001, η^2^_*p*_ = 0.56, with fewer errors committed in the singleton distractor absent condition (2.62%) than in the present condition (5.12%). However, neither the main effect of singleton distractor presence, nor the interaction between perceptual load and singleton distractor presence approached significance (both *F*s < 1), indicating that observers did not trade accuracy for speed.

Further evidence for the conclusion that the salient distractor had a different effect on visual search in the high load condition across Experiments 2A and 2B was sought by conducting a 2 × 2 mixed ANOVA on mean correct RTs with singleton distractor presence (present vs. absent) as the sole within-subjects variable and experiment (Experiment 2A vs. 2B) as the sole between-subjects variable. This analysis revealed a significant singleton distractor presence × experiment interaction, *F*_(1, 31)_ = 19.41, *p* < 0.001, η^2^_*p*_ = 0.39, indicating that the presence effect was significantly larger in the high load condition of Experiment 2A (208 ms) relative to the high load condition of Experiment 2B (16 ms).

Note that we also considered the possibility that the differential pattern of distractor presence effects observed across Experiments 2A and 2B might reflect the interplay between two processes: capture and practice. Some participants (those in the singleton distractor present first condition) had more practice than other participants (those in the singleton distractor absent first condition) when performing search on singleton absent trials. It is possible the large differences observed between the experiments are not solely due to differential processing of the singleton distractor, but the differences are so large because practice speeded response times for some participants and not others. Response time differences are our primary measure of distractor interference, and the change could be due to either slower responses in the presence of the critical distractor or a speeding of responses in the singleton absent trials. If search in the present study was subject to robust practice effects, we would expect to observer a significant difference in response times for the singleton absent trials between the two experiments. This analysis was performed on singleton absent trials and not singleton present trials because singleton present trials are potentially subject to differential processing of the critical distractor; singleton absent trials do not include the same confound. However, RTs during the singleton distractor absent trials were nearly identical in both Experiments 2A and 2B (*p* > 0.50) despite the increased practice the observers had when they encountered the distractor present blocks first (Experiment 2A).

### Discussion

**Figure 5:**
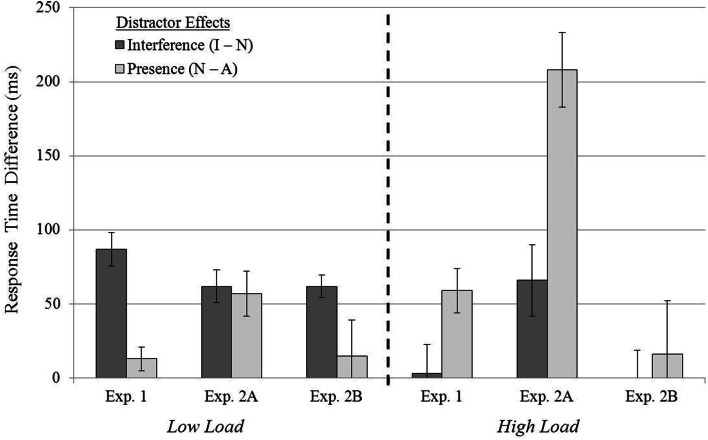
**Mean distractor interference (Incompatible − Neutral) and distractor presence (Neutral − Absent) effects shown as a function of perceptual load across experiments**. Error bars reflect the standard error of the mean.

In summary, Experiment 2B was identical to Experiment 2A with the sole exception that the observers did not encounter the salient distractor until the second (and fourth) trial blocks. However, contrary to the results of Experiment 2A, the results obtained in Experiment 2B suggested that perceptual load now dominated visual salience in the competition for visual selective attention. Singleton distractor compatibility analyses suggested that the incompatible distractor produced interference in the low load condition but not in the high load condition. Results of the presence effects corroborated this conclusion by suggesting that the presence of the singleton distractor did not cause a search-time cost in either load condition. This evidence suggests that the interplay between salience, dilution, and perceptual load in visual selection depends highly upon top-down factors such as task context and prior experience. We confirmed this conclusion by conducting a 3 × 2 repeated measures ANOVA with experiment (1, 2A, and 2B) as a between-subjects factor and singleton distractor condition (incompatible, neutral, or absent) as the within-subjects factor. See Figure 5 for results. The difference across experiments was non-significant for low perceptual load, *F*_(4, 100)_ = 1.74, *p* = 0.15, but the difference across experiments was significant for high perceptual load *F*_(4, 100)_ = 16.85, *p* < 0.001. Therefore, top-down factors have a much greater impact under high vs. low perceptual load conditions.

## General discussion

The present study provides several important contributions to our understanding of visual selective attention. First, by including two measures of distractor processing—singleton distractor compatibility and singleton distractor presence—the present study was able to provide two converging sources of evidence for its theoretical conclusions. This converging evidence was deemed necessary because recent developments have raised the possibility that the outcome of a single measure might not adequately disambiguate the various factors at work. Second, the present study also clarified the extent to which visual salience can dominate the control of focal attention during visual selection in this serial search task. Recall that previous studies using this paradigm had concluded that the dominance of visual salience over perceptual load was context-dependent (Biggs and Gibson, 2010). However, this pattern of dominance was puzzling given that the effect of salience appeared to become stronger in high load contexts despite increased knowledge of display characteristics. In contrast, other studies (see e.g., Lamy and Yashar, 2008; Müller et al., 2009; but see, Pinto et al., 2005) have typically shown that the effects of salience become weaker as knowledge of display characteristics increased. Contrary to these previous efforts, the present study attempted to manipulate context without altering the amount of display knowledge provided to observers. However, the critical manipulation under these circumstances appears to be the exposure or experience with singleton distractor absent trials. Findings obtained in Experiment 2A suggested that observers could not ignore the salient distractor, which could lead to the conclusion that visual salience dominated perceptual load. The findings obtained in Experiment 2B suggested that observers could ignore the salient distractor, which could lead to the conclusion that perceptual load dominated visual salience. The critical difference hinges upon blocking and prior exposure (or lack thereof) to the singleton distractor.

How might this relative ordering of singleton presence modulate the effects of a salient distractor? Current evidence suggests that the effect of salient distractors on attention can be modulated by the width of the attentional window, which in turn can be modulated by the type of search task observers are asked to perform (Gibson and Peterson, 2001; Theeuwes, 2004; Belopolsky et al., 2007). More specifically, the computation of visual salience typically requires the computation of a “difference signal” (Cave and Wolfe, 1990), which reflects the difference between each display item and all other display items along each feature dimension. Such computation appears to require that observers distribute their attention broadly across the display, such as when observers search for relevant singleton targets during singleton detection tasks (Theeuwes, 1991, 1992, 1994). After all, why would a red distractor pop-out from a homogenous green background if not for some processing of the background itself? Under these conditions, irrelevant singletons distractors typically capture attention so long as they are more salient than the relevant singleton targets (Theeuwes, 1991); though, there has been debate about whether the broad distribution of attention is sufficient for attentional capture, or whether it is also necessary for observers to engage in a particular type of feature processing strategy (e.g., singleton detection) while their attention is broadly distributed (for further discussion, see Bacon and Egeth, 1994; Lamy and Egeth, 2003; Theeuwes, 2004; Leber and Egeth, 2006a,b). In contrast, the visual salience of any given display item may not be identically computed when observers distribute their attention more narrowly on individual display items, such as when observers search for non-singleton targets during serial search tasks (Gibson and Peterson, 2001; Theeuwes, 2004; Belopolsky et al., 2007). Under these conditions, irrelevant singletons distractors typically do not capture attention (Jonides and Yantis, 1988; Folk and Annett, 1994).

The primary issue then becomes these two potential attentional allocation strategies that the observer might employ during search: broadly or narrowly tuned attention. On the one hand, observers might have an initial tendency to distribute their attention broadly across the display when the salient distractor is present, perhaps because they seek to first detect the singleton in order to subsequently avoid it during search. In so doing, they would utilize a search strategy that causes their attention to be captured by the salient distractor. Furthermore, once the salient distractor was located, observers would then need to distribute their attention more narrowly on individual display items within the relevant set in order to find the target. On the other hand, observers might have a tendency to only distribute their attention more narrowly on individual display items when the salient distractor would never be present.

In addition to the possibility that the distractor present and distractor absent conditions might be associated with two different attention allocation strategies, it is also possible that the attention allocation strategy used during a preceding block of trials might persist during a subsequent block of trials. Consistent with this notion, Leber and Egeth (2006a,b) have shown that observers who were forced to engage in one of two feature processing strategies—feature search or singleton detection—during an initial training phase continued to adopt that strategy during a subsequent test phase in which either strategy could have been used. Moreover, Leber and Egeth (2006a) also showed that the trained strategy was more likely to persist during the test phase following 320 training trials than following 40 training trials.

Similar to training effects, how observers distributed their attention during blocks of singleton distractor present trials may have depended on the amount of prior experience they had searching distractor absent displays. In other words, observers may have been more likely to distribute their attention narrowly during singleton distractor present trials as their exposure to singleton distractor absent trials increased. If so, then observers may not have relied as heavily upon locating the singleton distractor during a brief, but broadly tuned initial processing phase, leading to a reduction in attentional capture and ultimately distractor interference. Conversely, observers may also have been more likely to distribute their attention broadly during singleton distractor absent trials as their exposure to singleton distractor present trials increased. There would have been no observable consequences of distractor interference in this case as there was no singleton distractor present to provide interference, and we did observer similar overall response times for singleton distractor absent trials in both Experiments 2A and 2B. Furthermore, according to Leber and Egeth (2006a), the adoption of a particular attention allocation strategy becomes increasingly automated as task performance becomes increasingly associated with a particular attention allocation strategy. In this view, the present findings may be interpreted to suggest that observers' application of the narrow attention allocation strategy became more automatic as their exposure to the “training” (i.e., singleton distractor absent) task increased. Note, however, that whereas Leber and Egeth always presented blocks of training trials before blocks of test trials in their study, blocks of “training” (i.e., singleton distractor absent) trials were alternated with blocks of “test” (i.e., singleton distractor present) trials in the present study. Thus, the adoption of a broad attention allocation strategy could have competed with the adoption of a narrow attention allocation strategy in the present study because both could have been increasingly associated with task performance over time.

In conclusion, previous research has suggested that the competition between perceptual load and visual salience can be biased as a function of task context (Biggs and Gibson, 2010). Our present experiments extend this idea and provide an example of what may be included within the scope of “context.” Specifically, certain processing strategies may be experience-dependent and will lead the observer to adopting a narrow attentional set. This evidence supports an interpretation of attentional capture as a top-down effect. Therefore, even when capture appears to be dependent on salience, it can actually be the product of a processing strategy that prioritizes relative salience. The full extent to which an observer becomes biased toward a particular processing strategy, and the extent to which it transfers to different search displays, is a subject that requires further research.

### Conflict of interest statement

The authors declare that the research was conducted in the absence of any commercial or financial relationships that could be construed as a potential conflict of interest.
